# Design of 3D Controller Using Nanocracking Structure-Based Stretchable Strain Sensor

**DOI:** 10.3390/s23104941

**Published:** 2023-05-21

**Authors:** Seongjin Yang, Minjae Kim, Seong Kyung Hong, Suhyeon Kim, Wan Kyun Chung, Geunbae Lim, Hyungkook Jeon

**Affiliations:** 1 Pohang Accelerator Laboratory (PAL), Pohang University of Science and Technology (POSTECH), 77 Cheongam-Ro, Nam-Gu, Pohang 37673, Republic of Korea; seongjin@postech.ac.kr; 2 Department of Mechanical Engineering, Pohang University of Science and Technology (POSTECH), 77 Cheongam-Ro, Nam-Gu, Pohang 37673, Republic of Korea; mjkim@northwestern.edu (M.K.); skhong@postech.ac.kr (S.K.H.); kshyeon@postech.ac.kr (S.K.); wkchung@postech.ac.kr (W.K.C.); limmems@postech.ac.kr (G.L.); 3 Department of Physical Medicine & Rehabilitation, Northwestern University, 710 N. Lake Shore Dr., Chicago, IL 60611, USA; 4 Department of Manufacturing Systems and Design Engineering (MSDE), Seoul National University of Science and Technology (SEOULTECH), 232 Gongneung-Ro, Nowon-Gu, Seoul 01811, Republic of Korea

**Keywords:** strain sensor, motion analysis, stretchable materials, machine learning, human-machine interface

## Abstract

In this study, we introduce a novel design for a three-dimensional (3D) controller, which incorporates the omni-purpose stretchable strain sensor (OPSS sensor). This sensor exhibits both remarkable sensitivity, with a gauge factor of approximately 30, and an extensive working range, accommodating strain up to 150%, thereby enabling accurate 3D motion sensing. The 3D controller is structured such that its triaxial motion can be discerned independently along the X, Y, and Z axes by quantifying the deformation of the controller through multiple OPSS sensors affixed to its surface. To ensure precise and real-time 3D motion sensing, a machine learning-based data analysis technique was implemented for the effective interpretation of the multiple sensor signals. The outcomes reveal that the resistance-based sensors successfully and accurately track the 3D controller’s motion. We believe that this innovative design holds the potential to augment the performance of 3D motion sensing devices across a diverse range of applications, encompassing gaming, virtual reality, and robotics.

## 1. Introduction

Three-dimensional (3D) motion sensing is a crucial technology that identifies and analyzes the motion of objects to facilitate human-machine interfaces [1,2,3]. This innovative technology has been extensively applied in diverse domains, including medical diagnosis [4,5,6,7,8,9], virtual and augmented reality, kinetic motion measurement [10,11,12,13], robotics [14], industry, and education, each possessing significant market potential. Three-dimensional motion sensing techniques can be broadly classified into two primary categories: contact and non-contact methods. The contact approach involves the use of stretchable or wearable devices embedded with an array of sensors, such as inertial, skin conductivity, and sound pressure sensors [15,16,17,18,19,20,21,22,23]. In contrast, the non-contact method utilizes optical, ultrasonic, infrared [14], or magnetic fields [24,25,26,27,28] for motion detection. Depending on the user’s requirements, the parameters being measured, and the sensing environment, an appropriate method should be utilized for 3D motion sensing.

Although the non-contact method is particularly advantageous for accurately measuring overall movement without direct contact, it is susceptible to external factors, such as contrast and obstacles. Moreover, the method’s effectiveness is hindered when the medium used for measurement encounters obstructions. Consequently, the non-contact method is more appropriate for detecting motion in a specific space or environment rather than a moving object. Therefore, to assess 3D motion sensing without environmental restrictions, a contact method employing wearable motion sensing devices is a more adaptable approach.

In recent years, research on wearable devices has attracted significant interest, with the direct measurement employing stretchable or flexible strain sensors [29]. Although this method is constrained by its capacity to measure only specific aspects of movement where the sensor is attached, it surpasses other techniques in terms of sensitivity, and possesses the ability to measure various strains irrespective of location and direction, depending on the purpose [30,31,32,33,34,35]. Moreover, accurate measurement of diverse deformations, ranging from small (skin-level motions like pulsation) to large (joint-level motions) magnitudes, can be achieved with the appropriate sensor. The flexibility and diverse shapes and sizes of strain sensors also permit their attachment to the surface of non-flat, dynamic objects or an array of objects to measure the object’s deformation. Thus, 3D motion sensing can be conducted more effectively by using stretchable strain sensors customized for different structures and applications.

In previous work, we developed an innovative stretchable strain sensor based on the nanocracking structure, referred to as the omni-purpose stretchable strain sensor (OPSS sensor) [36]. In that work, we found a significant relationship between the thickness of a sputtered thin metal film and the nanocracking structure generated on the film under an externally applied tensile strain. By controlling and optimizing the nanocracking structure, we successfully developed the OPSS sensor, which exhibited both high sensitivity (gauge factor ~30) and an extensive working range (strain up to 150%) with excellent linearity (R^2^~0.98) and rapid response time (<30 ms) [36].

In this study, we designed a novel 3D controller by leveraging the capabilities of the OPSS sensor. The developed 3D controller features a flexible body equipped with multiple OPSS sensors at various positions to facilitate the quantification of deformations for 3D motion sensing. The controller’s flexible body was engineered to exhibit distinct deformation patterns when moved along each axis, with OPSS sensors strategically attached to these areas. Upon applying a 3D motion (or load) to the controller’s knob, the sensor affixed to the flexible body responds nearly independently along the corresponding axis. This response allows for the prediction of the applied 3D motion (or load) to the controller’s knob by assessing the change in the sensor’s resistance. Furthermore, to minimize interference between the sensors’ measurements and sensing errors induced by the material properties of the polymeric controller body, machine learning-based data analysis techniques were employed to compare resistance signals with actual knob movements and apply compensation based on the comparison, facilitating real-time and precise 3D motion detection. We argue that the developed 3D controller holds significant potential for application in fields such as medical surgical robotics and virtual reality, where precise and accurate movements are imperative.

## 2. Design and Methods

### 2.1. 3D Controller Fabrication

**Figure 1:**
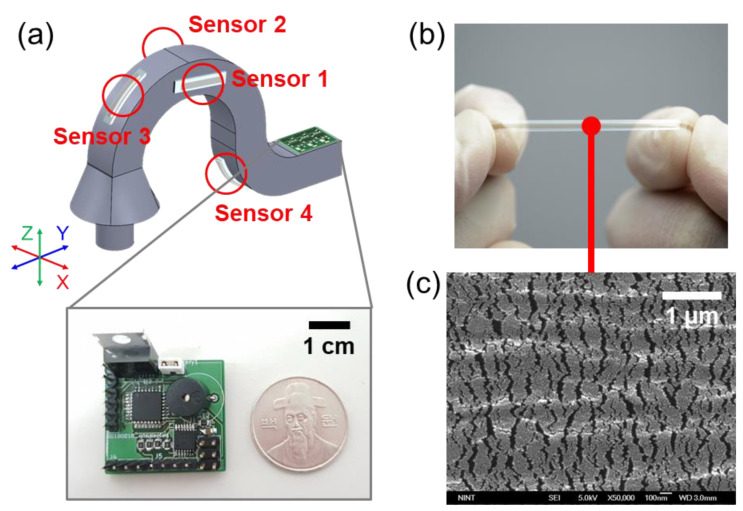
Overall schematic of 3D controller system. (**a**) The overall composition is divided into flexible body, OPSS sensor, and micro controller unit. (**b**) Flexible OPSS sensor (**c**) SEM image of pre-strained OPSS sensor.

As shown in Figure 1a, the developed 3D controller consists of a flexible body onto which multiple OPSS sensors have been attached at different locations to measure deformations for its 3D motion sensing. Through empirical design, the flexible body has been optimized to ensure that the controller’s knob can effectively assess externally imposed motions or loads.

The flexible body of a 3D controller should be able to move within an appropriate range when subjected to applied forces, while also being non-viscous to attach the OPSS sensor. To achieve this, poly(dimethylsiloxane) (PDMS) was used, and the detailed design can be found in Section 2.2. The device was simply fabricated by pouring the PDMS solution into a pre-fabricated acrylic mold, and to prevent the flexible body from sagging during operation due to weight, a 3D-printed skeleton made of PLA was added to the design before the PDMS casting process (see Supplementary Figure S1 for more details).

For measurement of 3D deformations of the flexible body, a stretchable strain sensor with both high sensitivity and wide sensing range is essential to enable precise measurement and increase working range of the controller (Figure 1b). For the purpose, we utilized the omni-purpose stretchable strain sensor (OPSS sensor) that was developed in our previous studies [36,37,38,39]. First, thermoplastic polyurethane (PU) beads (Pellethane 2363-80AE, Lubrizol, Wickliffe, OH, USA) were dissolved in a mixture of tetrahydrofuran (THF) and dimethylformamide (DMF) (60/40, *v*/*v*) to form a 10 wt% PU solution. Second, the prepared PU solution was used to make a PU membrane by spin-coating on a slide glass, controlling the thickness of the membrane through spinning speed (thickness of ~100 μm at 100 rpm), and dried in a 60 °C oven for approximately one day. Third, a thin layer of platinum (Pt) was deposited on the membrane by the magnetron sputtering method. Here, the sputtering time was empirically optimized to achieve both high sensitivity and wide sensing range at 50 s, which corresponds to ~10 nm of Pt thickness.

The real-time multi-resistance data of the 3D controller motion measurement system were measured using a PCB circuit equipped with a microcontroller unit (MCU) and a 12-bit analog-to-digital converter. The resistance measurement circuit can measure four resistance values in real time, and for this purpose, the MCP3204 12-bit A/D Converter (Quad Channel SPI Serial IC from Microchip Technology Inc., Chandler, AZ, USA), with a 12-bit resolution, was used to measure the reference voltage. The measured value of the circuit was converted into a resistance value through ATmega8L and 8 MHz MCU from Atmel. The size of the entire PCB circuit was 25 mm × 40 mm.

In our previous study [36], we found that the generation and propagation of nanocracks in the non-stretchable metallic (Pt) layer are the key mechanisms underlying the resistance change in the OPSS sensor. As demonstrated in the SEM image of the nanocracking structure generated on the platinum layer for the pre-strained OPSS sensor in Figure 1c, by empirically optimizing the Pt thickness, we can achieve a highly dense (crack density ≈ 10^7^/m) and nearly uniform-sized nanocracking structure, facilitating high linearity of resistance in response to externally applied strain [36]. During the initial loading on the sensor, nanocracks form on the Pt layer, and their width increases with increasing strain, resulting in an increase in resistance. Conversely, during the unloading stage, as the strain decreases, the width of the nanocracks decreases while their number is maintained, leading to a decrease in resistance. After the initial loading and unloading process, unless a strain higher than the initial loading one is applied, subsequent loading and unloading typically do not generate new cracks but rather change only the width of the existing cracks, resulting in a change in resistance.

For 3D motion sensing using the OPSS sensor, multiple sensors are required, and each sensor should detect both stretching and contracting modes at its respective location. To achieve this, each OPSS strain sensor was attached with 30% pre-strain applied, allowing the sensor to measure not only stretching but also contracting. If the sensor is attached to the 3D controller body without pre-strain, contraction at the corresponding location of the 3D controller body cannot be directly translated into a length change of the OPSS sensor. Instead, a gap would occur between the sensor and the surface of the 3D controller body. Consequently, we cannot obtain the resistance change of the sensor corresponding to the contraction accurately, and the induced gap could even cause damage to the sensor and detachment of the sensor from the 3D controller body. By applying pre-strain when attaching the OPSS sensor to the surface of the 3D controller body, we can address this issue, as we can analyze the contraction from the resistance decrease. The contraction would cause the crack width to decrease from the originally expanded width due to the pre-strain, similar to a typical unloading process. This approach enables the measurement of not only stretching but also contraction at the attachment site, resulting in more accurate device motion measurements using fewer sensors.

### 2.2. Numerical Analysis of Deformation of the Flexible Body

Due to the constraint that only deformations at the sensor attachment points can be measured, determining the optimal sensor placement on the flexible body of the controller is critical for accurately assessing the 3D motion of the controller’s knob. Ideally, each sensor would separately evaluate the motion of each axis, enabling a direct translation of individual sensor measurements to the corresponding axis motion without the need for further decomposition of multiple measurements.

However, obtaining perfectly axis-separated measurements from sensors affixed to a single-body structure is not feasible, as all sensor readings are inherently interdependent. Despite this limitation, it remains essential to optimize the sensor attachment positions to achieve high sensitivity and efficient decoupling of 3D motion. In order to identify the most suitable locations, we conducted a numerical analysis of the flexible body’s deformation under three-axis loads, utilizing COMSOL Multiphysics for the simulations.

**Figure 2:**
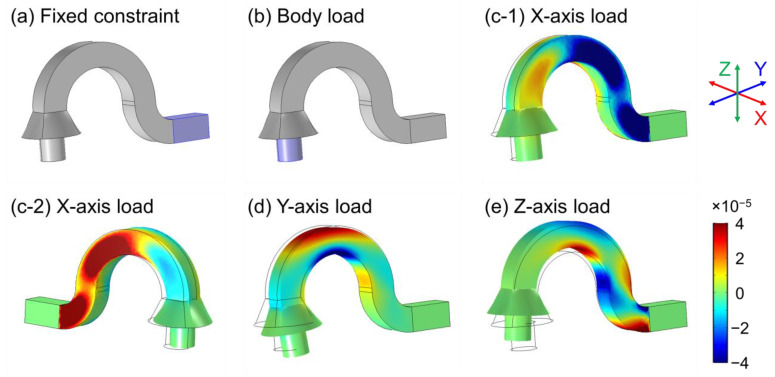
Numerical analysis of 3D deformation caused by the multi-axis motion of the designed flexible body. The blue highlighted regions indicated the regions where we applied (**a**) fixed constraint and (**b**) external body load boundary conditions. (**c**–**e**) represent the magnitudes of Y-axis normal strain (ε_yy_) under the X-, Y-, and Z-axis body load conditions (at 0.01 N), respectively. The black solid lines in (**c**–**e**) represent the original location of the flexible body before deformation.

The flexible body’s geometry was designed using 3D CAD software (SolidWorks 2018) and subsequently imported into the numerical simulation software. The Solid Mechanics module was utilized in the numerical analysis, with two key boundary conditions applied: a “Fixed constraint” condition to designate one end of the flexible body as fixed, and a “Body load” condition to apply external motion (or load) to the knob of the flexible body, as depicted in Figure 2a,b. The optimized sensor position should satisfy two requirements: high deformation and minimal interference between sensors. As the sensors are attached to the flexible body in the longitudinal direction, which corresponds to the Y-axis in Figure 2, we analyzed the magnitudes of Y-axis normal strain (εyy) under X-, Y-, and Z-axis body load conditions (at 0.01 N), respectively. Figure 2c–e illustrate the magnitudes of Y-axis normal strain (ε_yy_) under X-, Y-, and Z-axis body load conditions, respectively. Although it is not feasible to identify specific regions affected exclusively by one axial load independent of other axial loads, we were able to select regions predominantly influenced by each axial load with high deformation. The optimized sensor positions were determined primarily based on numerical analysis results with sensor attachment compatibility. In order to measure both stretching and shrinking modes at specific regions using an OPSS sensor, it is necessary to apply pre-strain to the sensor when affixing it to the flexible body’s surface. Consequently, a convex-shaped surface is more compatible for sensor attachment. The determined optimized sensor positions are represented in Figure 1a, where sensors 1 and 2 are primarily responsible for the X-axis motion (or load) of the knob of the flexible body, while sensors 3 and 4 predominantly account for the Y- and Z-axis motion (or load), respectively.

### 2.3. Machine Learning Based Estimation of 3D Controller Position

To obtain the 3D position of the proposed controller and identify the relationship between the resistive sensor data and 3D motion, we utilized Leap Motion (Ultraleap, Bristol, UK). The ground-truth position of the controller was determined by using the 3D position of the distal bone of the thumb that pinches the proposed controller. The resistive sensor data calculated by the MCU were transmitted to a personal computer via serial communication. We collected both the resistive sensor data and Leap Motion data simultaneously every 0.1 s and used a third-order low pass filter with a 0.5 Hz cut-off frequency to remove noise interference. Then, we used a shallow neural network trained using Levenberg–Marquardt backpropagation [40], to establish the relationship between the four resistance signals and the 3D position data (i.e., x, y, and z position). The MATLAB-built-in fitnet function was utilized for this purpose with default parameters including one hidden layer of size 10, a learning rate of 0.001, decrease factor for learning rate of 0.1, and increase factor for learning rate of 10, respectively.

For training data collecting, we manually controlled the controller in an arbitrary manner, taking care to avoid any occlusion of the Leap Motion. After each trial, we maintained the final position of the controller for one second before returning to its starting point. More than 5 trials were conducted, and the data were collected for training. To evaluate the performance of the proposed method, we calculated the coefficient of determination (or R^2^ score).

## 3. Results and Discussion

### 3.1. Independent Resistance Change Measurement for Each Axis and Visualization

**Figure 3:**
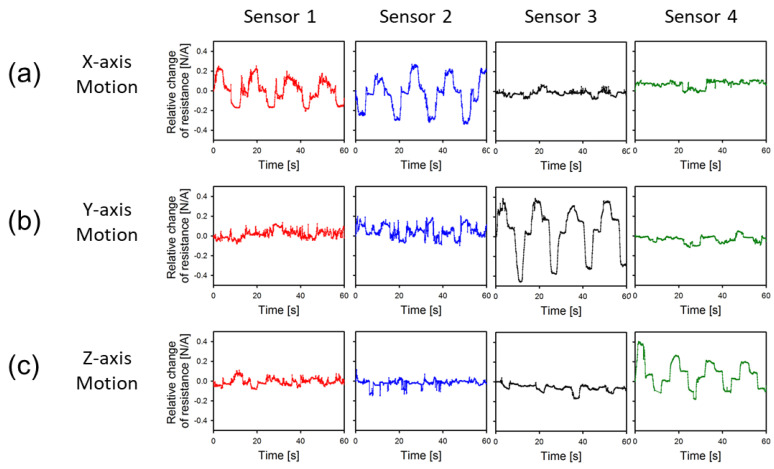
Resistance change of each sensor measured when moved in (**a**) X-, (**b**) Y-, and (**c**) Z-axes.

In this study, we evaluated the device’s performance by attaching the OPSS sensor to the flexible body of the 3D controller. As the knob of the 3D controller moves, the flexible body also moves, causing deformation in specific parts that correspond to each axis. By attaching the OPSS sensor to these deformation parts, the movement of each axis can be accurately measured. However, to ensure precise measurement of the movement, it is essential that the change in resistance of the sensor is independent of the movement in each axis. For instance, when the 3D controller moves along the X-axis, the resistance of the sensor attached to the deformed part of the X-axis changes, while the resistance of the sensors attached to the deformed parts of the Y-axis and Z-axis must remain unchanged. To verify this, we conducted a deformation test by attaching the sensor to the corresponding positions on the 3D controller body determined in the numerical analysis and then moving the knob of the 3D controller along each axis. The resulting resistance changes for the X-axis, Y-axis, and Z-axis movements are shown in Figure 3a–c, respectively; in Figure 3, graphs show the profiles of the ‘relative change of resistance’ which is defined as $(R_{on}-R_{off})/R_{off}$. As a result, when the knob moved in the X-axis direction, the +X and -X sensors showed resistance changes in opposite directions, while there was little change in resistance for the Y-axis and Z-axis sensors. This suggests that the attached sensor can measure tension and contraction well, and the resistance changes nearly independently for different axes. Similarly, we observed similar resistance changes for the remaining sensors in the Y-axis and Z-axis movements.

Through these observations, it was verified that only the sensors corresponding to the direction of motion were dominantly activated, featuring almost independent deformable parts along each axis. Furthermore, it is feasible to measure both tension and contraction strain of each axis through the pre-strained method, signifying that the target’s movement can be detected instantaneously and accurately regardless of the deformation mode. If the knob moved along a specific axis, the resistances of sensors responsible for the other axes exhibited minimal changes; however, even these small fluctuations can significantly impact motion detection accuracy. A detailed discussion of this error can be found in Section 3.2.

**Figure 4:**
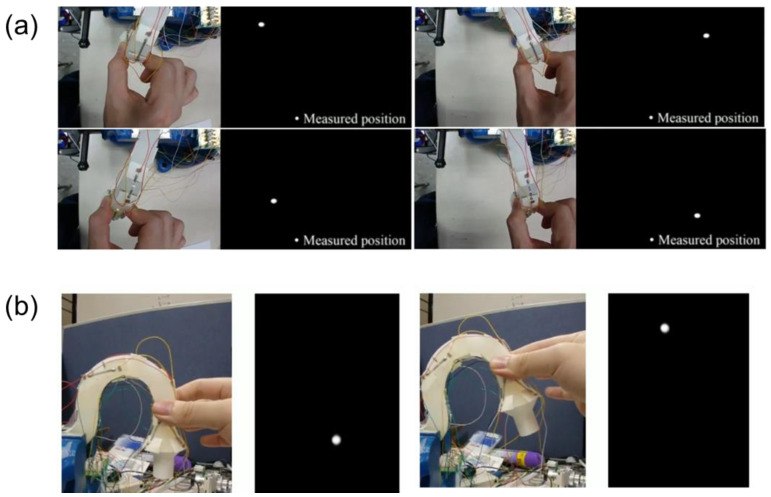
Single-axis strain measurement test and data visualization. Motions on (**a**) XY plane and along (**b**) Z-axis.

The independent changes in resistance data for each axis when the 3D controller is moved imply that not only simple movements of the X-, Y-, and Z-axes but also complex movements can be accurately displayed. Furthermore, if these resistance data are collected in real time and simultaneously, the movement of the 3D controller can be traced back with only the resistance data. In this regard, Figure 4 shows the result of visualizing the movement of the 3D controller based on the measurements of four OPSS sensors attached to the optimized locations of the controller. Four OPSS sensors were attached to the deformation part of each axis of the 3D controller, and the resistance change of each axis was measured in real time through the MCU-based real-time multi-resistance measuring system. First, after attaching sensors to the deformation parts of the X- and Y-axes, the results of predicting the position of the knob through the resistance data change when the 3D controller knob is moved in the order of upper left, upper right, lower left, and lower right on the XY plane are presented in Figure 4a. At this time, it was confirmed that the movement of the knob was accurately measured because the movement of each axis did not affect each other’s resistance data even in the complex movement in which the X and Y components were mixed. Therefore, the predicted position matched the actual position accurately. Similarly, the position of the knob can be predicted by collecting the change in resistance when the knob is moved in the Z-axis direction, as shown in Figure 4b. In addition, the sensor was attached to the Z-axis deformed parts and the movement of the knob was successfully sensed and visualized. When the 3D controller moves, the position can be expressed as X, Y, and Z components by the Cartesian coordinate system, and the final position is determined by the sum of each movement. Likewise, the sum of the corresponding resistance changes on each axis can accurately track the position of the 3D controller.

In order to confirm a slightly more complicated movement, a device that implements the same movement as a remote control was produced. To this end, resistance data produced when the sensor-attached 3D controller moved were collected, and the system was designed to represent it as position data in the MATLAB environment and implement the same movement through position control of the robot arm. As a result, it was confirmed that the movement of the 3D controller was normally converted into data form, and that the robot arm moved to the same position as the movement of the 3D controller. Detailed results may be found in Supplementary Video S1.

### 3.2. Compensation of Limitations of Polymers through Data Learning

**Figure 5:**
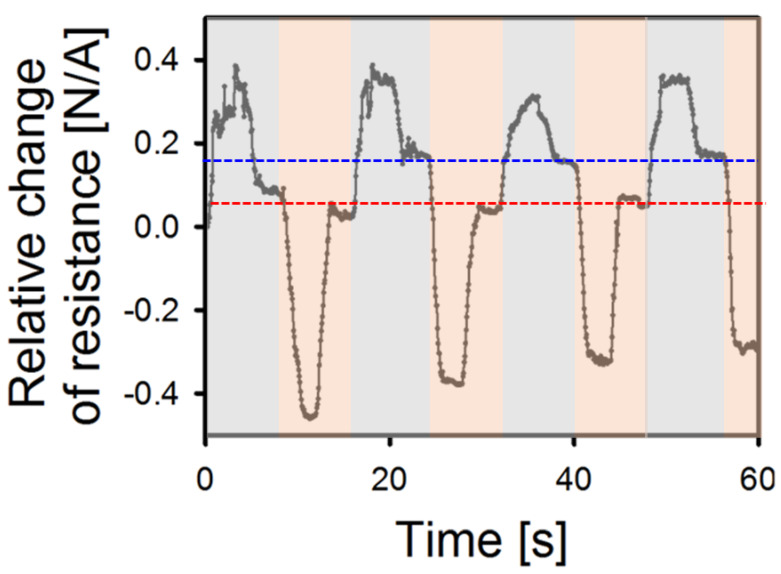
Resistance change of sensor and two rest resistance in front and back motion; the blue and red dotted lines represent the tension and contraction phases, respectively.

In the previous section, it was demonstrated that the sensors attached to the 3D controller can track its movement along the corresponding axis with a good accuracy. Although the resistances of the sensors accountable for other axes did not exhibit substantial changes,, even minor alterations can significantly degrade motion detection accuracy. In addition, the material characteristics of the sensor should be considered, as they directly affect the applied strain to the OPSS sensor. Figure 5 shows the change in resistance when the sensor corresponding to the Y-axis strain attached to the flexible body of the 3D controller is repeatedly moved in the +Y and −Y directions. At this time, different residual resistances are shown when returning to the initial position in the + and − directions. This phenomenon, which is similar to the backlash of mechanical parts, is a typical problem when polymers are stretched, and these properties are particularly strong in polymers under high elongation conditions. This can be explained by the viscoelastic properties inherent to elastomeric materials, such as flexible polymers. The viscoelastic behavior of elastomers is known to influence the stress-strain relationship, generating a hysteresis loop due to cross-linking during repetitive tensile loading at a constant strain [41]. Specifically, when an external force is exerted upon a viscoelastic polymer, the force is not instantaneously propagated through the cross-linked network, as would be the case for an ideally elastic body. Instead, it behaves as if temporarily stored within the polymer matrix. This leads to a nonlinear response during both tension and compression phases of the polymer. Consequently, the stress softening effect and imperfect elastic recovery become apparent. As a result, under identical stress conditions, the strain experienced by the material will vary according to the path of tension and compression.

Figure 5 shows an experiment in which a 30% pre-strained sensor was attached and repeated tension was applied in the +Y and −Y directions. At this time, the attached sensor is stretched 30% in the initial state, but after stretching and rest in the +Y direction, it does not exactly return to the initial state, 30% tension. According to the viscoelastic effect of elastomer, it actually stops at a higher strain state due to the viscoelastic effect. Likewise, when returning to the initial state of 30% tension after contraction in the −Y direction, it stops at a strain lower than 30% due to the delay in elastic recovery. As a result, the resistance change curve appears different due to the strain recovery delay effect during tension and contraction, and two residual resistances occur. That is, measuring tension and contraction with a single sensor causes a serious problem of low accuracy.

The results presented in Figure 5 demonstrate the behavior of the resistance of the Y-direction stretchable sensor when the 3D controller undergoes a repeated Y-direction front/back motions. Specifically, they show change in resistance in the tension/contraction cycle and the recovery phase of the polymer after each cycle. Tension and contraction were performed in the light blue and orange boxes, respectively. It was observed that two residual resistances were generated, as shown by the blue dotted line in the tension phase and the red dotted line in the contraction phase. This phenomenon indicates that the sensor cannot accurately measure the position data when the direction of motion is changed. The cause of this phenomenon is attributed to the viscoelastic properties of polymers, where the polymers are connected in a crosslinking structure and resulting elastic recovery delay effect. When the polymer is stretched, the crosslinking structure of the polymers is released, and the polymers crosslink with each other at the stretched state. When tension is applied in the initial state, the resistance deformation is not large until the crosslinked structure is broken, but after being broken, the resistance change increases rapidly. Similarly, when restored to the original state after applying tension, due to being crosslinked in the tensile state, residual resistance is observed at a resistance slightly higher than the initial resistance after tension restoration because of the stress softening effect. This problem also occurs in the contraction phase, and consequently, two different paths are made in the stress-strain curve when contraction and tension are repeated, rather than a straight path. Therefore, additional solutions are needed to overcome the material limitations of these polymer sensors under high elongation conditions and the induced error.

**Figure 6:**
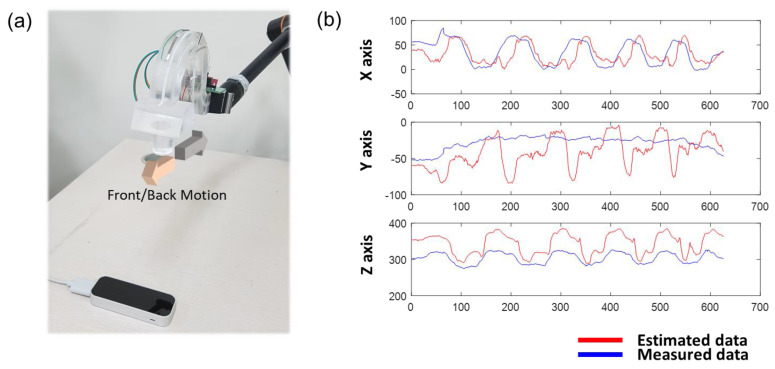
Position comparison using sensor recognition system and position prediction using sensor. (**a**) Motion recognition by Leap Motion when 3D controller has front and back motion (**b**) Comparison of front-back motion predicted through machine learning and sensors and Leap Motion position tracking.

This section aims to tackle the challenges stemming from the inherent limitations of polymers by utilizing machine learning techniques, as opposed to relying solely on material-based solutions. Although there have been previous studies in which machine learning was employed for motion detection, questions remain regarding its stability and reliability, and most of these prior works have primarily focused on measuring unidirectional motion rather than multi-axis movements [42,43,44]. To this end, resistance sensors were attached to the X, Y, and Z axes of the 3D controller to measure the resistance change, while the motion of the 3D controller was visually captured through the Leap Motion camera (i.e., the thumb’s trajectory) and location tracking device. As shown in Figure 6a, the position data of the 3D controller were captured through the Leap Motion camera and the resistance was measured through the sensor attached to the 3D controller. In addition, by learning the position data of the Leap motion from the resistance data obtained by the sensor, the position of the 3D controller can be tracked only with the resistance data. In addition, we checked whether meaningful results can be obtained in practice from the learning algorithm produced in this way. At this time, although the OPSS sensor has a rapid response time(<50ms), and Leap motion can operated with a higher sampling rate than 100 Hz, we limited the sampling rate to 10 Hz for 3D position estimation. This was to minimize the crosslinking problem of the material, as a higher sampling rate tended to degrade performance for the simple neural network we used. Implementing a network considering temporal characteristics of sensors, such as Long short-term memory [45], will facilitate more accurate estimation with a higher sampling rate.

Figure 6b is the result of comparing the position data of the 3D controller obtained through the Leap Motion with the position data derived only with resistance change through the learning algorithm. As a result, inaccurate or non-measurable results were obtained. In the graph, the red line is the position data value measured by the Leap Motion, and the blue line is the position data value derived through the learning algorithm when there is a change in resistance. Although the position is matched for the X-axis, the position data value is not derived at all for the remaining Y-axis, and in the case of Z, the movement is smaller than the actual movement. It can be confirmed that the position data cannot be tracked with only a sufficient resistance. This is the crosslinking problem of polymer materials mentioned above, and it that occurs because the resistance value continues to change even at the same location. Even when the resistance pointing to the position 0 is set through the learning algorithm, the same position shows different resistance according to the tension and contraction of the 3D controller, which becomes an obstacle in tracking the movement of the 3D controller.

This appears to be the case because the crosslinking of the polymers mentioned above affects the tension and restoration, and it seems to be the result of learning data from which different resistance results were derived at specific locations, due to the viscoelastic effect, by crosslinking during learning. In particular, the degree of crosslinking in the initial zero tension and in the tension state is different, and in the previous algorithm, all resistance changes were learned while ignoring this. However, in order to predict the motion more accurately, the resistance change in the stationary state before tension was excluded from the learning algorithm, and only the change in resistance during tension was applied to the learning algorithm, to learn only at the same degree of crosslink.

**Figure 7:**
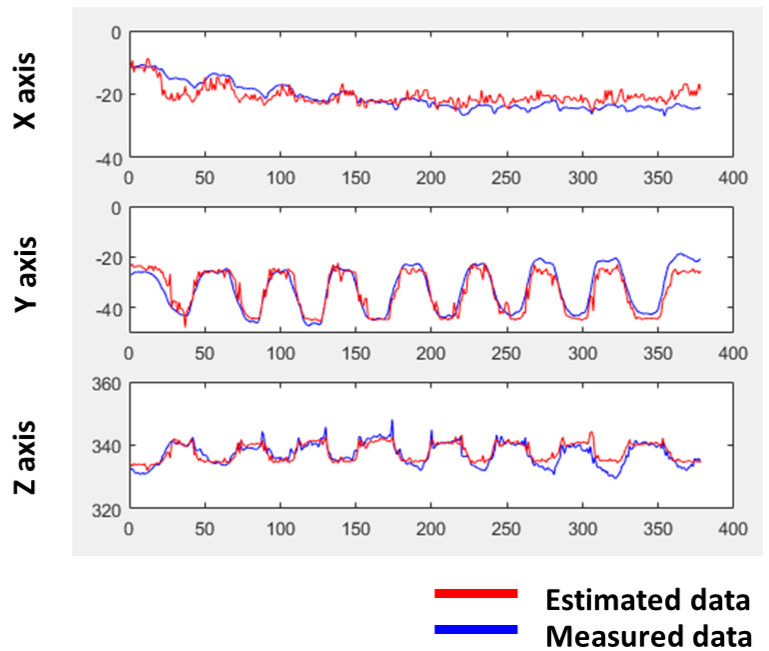
Position comparison using sensor recognition system and position prediction using sensor after changing the learning range.

As shown in Supplementary Figure S2, it was observed that the resistance of the sensor is similar at the same position when tension or contraction is repeated, except that the resistance when the tension starts is about 4% lower. This is considered to be because the polymers form crosslinks before starting the tension, and the resistance change is small when the polymers are stretched until the crosslinks are broken. Accordingly, the learning was attempted by omitting the resistance data of the stationary state. From the results, we found that accurate position prediction can be achieved by comparing the algorithm learned from the second repetition and after tension repetition with the actual position data by Leap motion. Upon comparing the algorithm’s learning results from the second tensile iteration with the actual position data, it was demonstrated that a relatively accurate position prediction could be achieved, exhibiting a coefficient of determination (or R^2^ score) of 0.8878, with the exception of the first tensile event (Figure 7). The findings demonstrate that 3D motion can be predicted across an extensive deformation range and intricate repeated motions in multiple axes using multiple sensors, compared to the previous studies, where machine learning was primarily employed for calibrating sensor performance in relatively small movements and repetitions of unidirectional motion using a single sensor [42,43,44].

## 4. Conclusions

In this study, a new type of 3D controller was developed, employing the OPSS sensor to measure its 3D motion. A numerical simulation was conducted to optimize sensor placement on the flexible body of the 3D controller, with the aim of achieving efficient and precise quantification of 3D motions in the X, Y, and Z axes. By attaching OPSS sensors to the optimized locations, interference between the measurements of sensors on each axial motion was minimized, ensuring that only the sensor connected to the deformed segment of the moving axis responded, predominantly autonomously, during motion along a specific axis. This enabled the accurate quantification of intricate movements as three variables, while simultaneously achieving high sensor sensitivity to input motion. Furthermore, machine learning algorithms were employed to address the material limitations of residual strain and incomplete spontaneous deformation recovery in polymer-based sensors by acquiring and processing the actual motion through Leap Motion technology and the resistance changes resultant from attaching the OPSS sensor. We anticipate that the developed 3D controller can exert a widespread influence across numerous applications, including medical robotics, wearable devices, motion monitoring systems, and various industrial sectors.

## Data Availability

The datasets generated during and/or analyzed during the current study are available from the corresponding author on reasonable request.

## Supplementary Materials

The following supporting information can be downloaded at: https://www.mdpi.com/article/10.3390/s23104941/s1, Figure S1: Fabrication of the flexible body part of the designed 3D controller.; Figure S2: Comparison of different initial resistances in X-axis tensile repetitions; Video S1: Control of a robot arm using the developed 3D controller.
