# Supplementary material for: Design of 3D Controller Using Nanocracking Structure-Based Stretchable Strain Sensor
Source: Sensors (Basel). 2023 May 21;23(10):4941. doi: 10.3390/s23104941 (PMC10223148; doi:10.3390/s23104941)
Supplement: Supplementary file 1 [file sensors-23-04941-s001.zip › Supplementary Information_rev.pdf]

- Supplementary Information -

Design of 3D Controller Using Nanocracking Structure-Based Stretchable Strain Sensor

Seongjin Yang[1,2] , Minjae Kim[2,3], Seong Kyung Hong[2], Suhyeon Kim[2], Wan Kyun Chung[2], Guenbae Lim[2], and Hyungkook Jeon[2,4,]*

[1]Pohang Accelerator Laboratory(PAL), Pohang University of Science and Technology (POSTECH), 77 Cheongam-Ro, Nam-Gu, Pohang 37673, The Republic of Korea

[2]Department of Mechanical Engineering, Pohang University of Science and Technology (POSTECH), 77 Cheongam-Ro, Nam-Gu, Pohang 37673, The Republic of Korea

[3]Department of Physical Medicine & Rehabilitation, Northwestern University, 710 N. Lake Shore Dr., Chicago, IL 60611, USA

[4]Department of Manufacturing Systems and Design Engineering (MSDE), Seoul National University of Science and Technology (SEOULTECH), 232 Gongneung-ro, Nowon-gu, Seoul 01811, the Republic of Korea

*Corresponding authors:

hkjeon@seoultech.ac.kr (H. Jeon)

Table of Contents

Supplementary Figure S1. Fabrication of the flexible body part of the designed 3D controller. (a) the flexible body without a skeleton exibits pre-deformation due to its weight. (b) A schematic and (c) a picture of the mold design for the flexible body embedding a skeleton to minimize the pre-deformation. (d) a schematic of the flexible body embedding a skeleton.

Supplementary Figure S2. Comparison of different initial resistances in X-axis tensile repetitions in an integrated system with an OPSS sensor attached. The initial resistance of the first tension was about 200 ohms lower than the other initial resistances.

Supplementary Video S1. Control of a robot arm using the developed 3D controller. The resistance information of the integrated system attached with the OPSS sensor is converted into MATLAB and learning data, and the robot arm moves according to the measured movement.

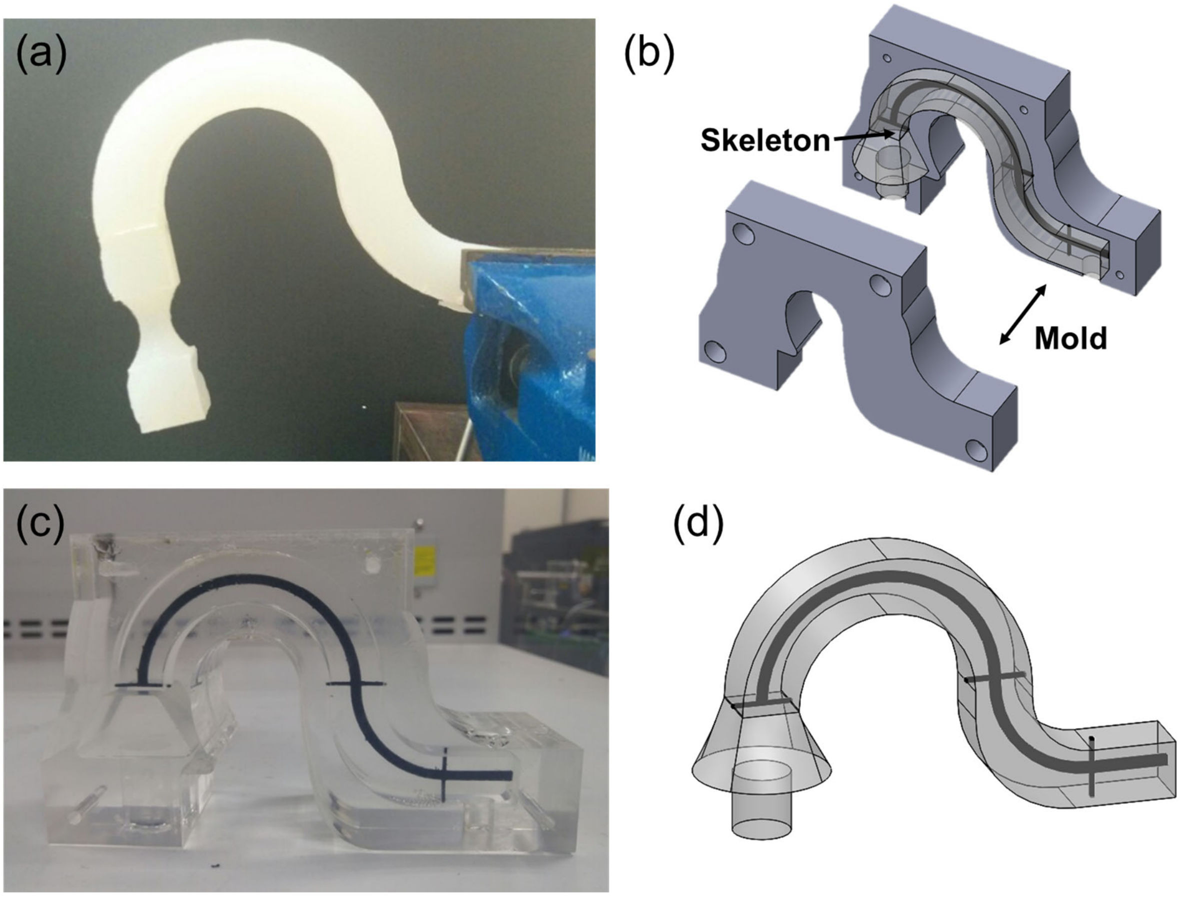

Supplementary Figure S1. Fabrication of the flexible body part of the designed 3D controller. (a) the flexible body without a skeleton exibits pre-deformation due to its weight. (b) A schematic and (c) a picture of the mold design for the flexible body embedding a skeleton to minimize the pre-deformation. (d) a schematic of the flexible body embedding a skeleton.

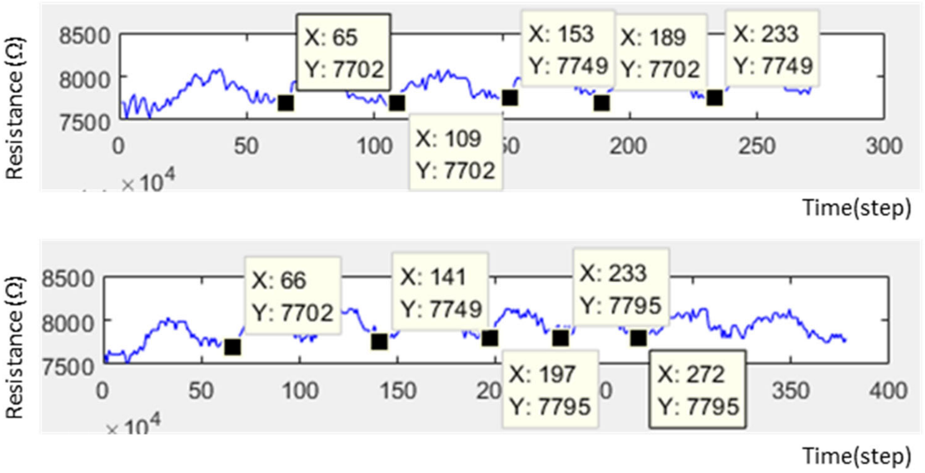

Supplementary Figure S2. Comparison of different initial resistances in X-axis tensile repetitions in an integrated system with an OPSS sensor attached. The initial resistance of the first tension was about 200 ohms lower than the other initial resistances.
